# Change in bias in self-reported body mass index in Australia between 1995 and 2008 and the evaluation of correction equations

**DOI:** 10.1186/1478-7954-9-53

**Published:** 2011-09-25

**Authors:** Alison J Hayes, Philip M Clarke, Tom WC Lung

**Affiliations:** 1 Sydney School of Public Health, University of Sydney, Sydney NSW 2006, Australia

**Keywords:** Obesity, self-reporting bias, BMI, correction equations

## Abstract

**Background:**

Many studies have documented the bias in body mass index (BMI) determined from self-reported data on height and weight, but few have examined the change in bias over time.

**Methods:**

Using data from large, nationally-representative population health surveys, we examined change in bias in height and weight reporting among Australian adults between 1995 and 2008. Our study dataset included 9,635 men and women in 1995 and 9,141 in 2007-2008. We investigated the determinants of the bias and derived correction equations using 2007-2008 data, which can be applied when only self-reported anthropometric data are available.

**Results:**

In 1995, self-reported BMI (derived from height and weight) was 1.2 units (men) and 1.4 units (women) lower than measured BMI. In 2007-2008, there was still underreporting, but the amount had declined to 0.6 units (men) and 0.7 units (women) below measured BMI. The major determinants of reporting error in 2007-2008 were age, sex, measured BMI, and education of the respondent. Correction equations for height and weight derived from 2007-2008 data and applied to self-reported data were able to adjust for the bias and were accurate across all age and sex strata.

**Conclusions:**

The diminishing reporting bias in BMI in Australia means that correction equations derived from 2007-2008 data may not be transferable to earlier self-reported data. Second, predictions of future overweight and obesity in Australia based on trends in self-reported information are likely to be inaccurate, as the change in reporting bias will affect the apparent increase in self-reported obesity prevalence.

## Background

The increasing prevalence of obesity is a major public health concern in most developed countries throughout the world. The most common means of determining obesity in population studies is through the use of body mass index (BMI) determined from weight in kilograms divided by the square of height in meters [1]. Frequently, this is based on self-reported height and weight data, which are necessary in postal or telephone surveys, or because it is impractical or too costly to take actual measurements. However, numerous studies have found that self-reported data tend to overestimate height and underestimate weight [2], leading to an underestimation of BMI and the proportions of overweight and obesity. It has also been suggested that use of self-reported data may bias the association of BMI with mortality [3].

While many studies have documented the bias in height and weight from self-reported data, few studies have examined the determinants of the bias or whether the bias has changed over time. A recent North American study found that the discrepancy among people in the United States has remained relatively constant between 1976 and 2004, while in Canada the discrepancy has increased over the period 1986 to 2005 [4].

Possibilities for adjusting BMI based on self-reported data include use of different BMI cut-off points [5,6] or adjustment of BMI estimates through the use of appropriate correction equations applied to self-reported height and weight [7,8]. While the use of lower BMI cut-points is a simple method, it cannot account for variations in the error across the population. For example, previous research in Australia [7] and other parts of the world [8-10] has shown that reporting error depends on both age and sex of the respondent. Hence, correction models including such factors as covariates will have greater likelihood of accurately adjusting self-reported data across different population subgroups.

In 2007-2008, the National Health Survey (NHS) undertaken by the Australian Bureau of Statistics (ABS) collected for the first time exact measures of height and weight in addition to self-reported data [11]. This survey follows a similar comparison of measured and self-reported data conducted by the ABS in 1995 using two different surveys [12]. It has enabled us to examine whether the bias associated with self-reported data has changed in Australia in the last 12 years and to derive correction equations based on the most recently available data.

The aims of this study are threefold: (i) to examine whether bias in reporting of anthropometric data has changed in Australia between 1995 and 2008, (ii) to investigate determinants of the bias, and (iii) to derive correction equations, which can be applied to self-reported data to adjust for the reporting bias.

## Methods

### Study population

Three nationally representative health surveys from Australia, conducted by the ABS, provided the data in this study. They were the National Nutrition Survey (NNS) in 1995 and NHS in 1995 and 2007-2008. The NHS are cross-sectional surveys carried out every three to four years using a stratified multistage area sample design [11,13]. They include private dwellings in urban and rural areas across all states and territories of Australia. After randomly sampling households, individuals were interviewed by trained personnel within the home. The overall response rates were over 90% for both the 1995 and 2007-2008 NHS [11,13]. In the 1995 NHS, only self-reported height and weight were collected, but a random subsample of respondents who agreed to take part in the NNS had their height and weight accurately measured two to three weeks later. The 2007-2008 NHS measured exact height and weight of all consenting participants; it also included questions on self-reported height and weight, which were posed as part of the same interview before exact measurements were taken.

### Data

Survey participants were asked to report their height and weight without shoes; if these data were supplied as an imperial measurement, they were converted to a metric measurement. Height was measured using a stadiometer and recorded in centimeters correct to two decimal points. Weight was determined using digital platform scales and recorded in kilograms correct to one decimal point. Reporting error was calculated as the difference between self-reported and measured values for height (cm), weight (kg), and BMI (i.e., self-reported minus measured values).

De-identified data were provided by the ABS as confidential unit record files (CURFs) [13,14]. Continuous measures of height between 145 cm and 199 cm and of weight between 40 kg and 139 kg were available in the CURFs. Measured and self-reported heights and weights outside these ranges were reported as a category and hence could not be used. Study data were derived similarly from both 1995 and 2007-2008 surveys. We excluded from the analysis people with missing height or weight (measured or self-reported) and pregnant women. Outliers - defined as persons with reporting error of ± 4 standard deviations from the mean - were also omitted from the analysis. For comparability with the 1995 survey, in which 20 to 24 years was the youngest adult age group, we restricted the analysis to adults over the age of 20 years. The study dataset from 1995 included 9,635 persons aged 20 years and over; in 2007-2008 the study dataset comprised of 9,141 people.

### Statistical analysis

Summary statistics of BMI based on reported and measured height and weight were determined, and a graphical analysis was undertaken to examine the relationship between reporting bias and age and between reporting bias and measured height or weight across the two surveys.

In a main effects multivariate linear regression model, we investigated significant determinants of the reporting bias in BMI using available demographic and socio-economic predictor variables. The dependent variable used in our analysis was the difference between self-reported and measured BMI and explanatory variables, including measured BMI, sex, age in years, deciles of socio-economic index for area (SEIFA), and dichotomous variables for being born in Australia, living in an urban environment, completion of year 12 of school, and possession of a tertiary qualification.

All analyses were carried out with the survey estimation commands of STATA v11.0 [15] using individual person weights, which account for the stratified sampling design and allow estimates to be representative of the Australian population. Standard errors (SE) and 95% confidence intervals (CI) were determined using a jackknife estimation method [16] using the 60 replicate weights provided in the NHS.

### Correction equations

In order to maximize the utility of correction equations, we focused on inclusion of information typically available in surveys and covariates already known to affect reporting bias, namely age, sex, and self-reported height, weight, and BMI. We investigated two different methods for predicting actual BMI from self-reported data. First, we derived separate correction equations for height and weight, and second, we derived one correction equation to directly adjust self-reported BMI. Survey regression analysis was employed, where the dependent variable was the reporting error in height, weight, or BMI, and independent variables were age, sex, and the relevant self-reported variable. As the graphical analysis revealed variation in the pattern of misreporting among men and women, models were initially fitted separately for men and women and only combined if there were no significant differences in any of the coefficients. Covariates were dropped from the regressions if they did not achieve significance at p < 0.05.

## Results

### Descriptive statistics

**Figure 1:**
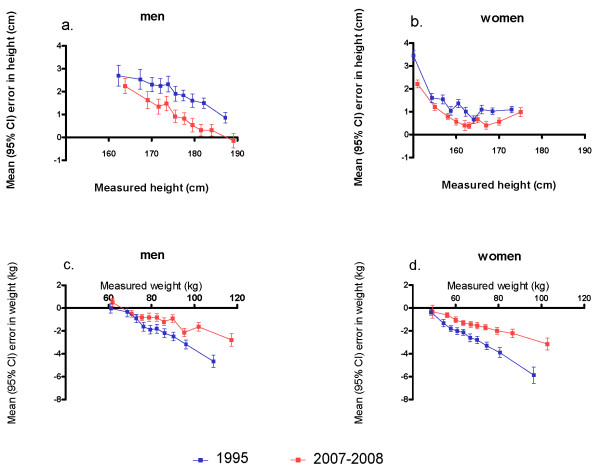
**Reporting error in height and weight (population-weighted) for men and women by deciles of measured height and weight, determined from Australian NHS in 1995 and 2007-2008; a. height error in men; b. height error in women; c. weight error in men; d. weight error in women**.

Table 1 shows that BMI using self-reported data was underestimated across all male and female age groups in both 1995 and 2007-2008, but that the magnitude of the bias has diminished between the two surveys. The underestimation of BMI is due to both the overreporting of height and the underreporting of weight; however, the accuracy of reporting height and weight has improved across all sectors of the adult population of Australia between 1995 and 2007-2008 (Figure 1). Among men, there is a negative linear relationship between height error and measured height, with shorter men overestimating their height by larger amounts. Reporting of height was most accurate among men in the tallest decile of height, with the mean reporting error close to zero in 2007-2008. For women, the pattern of misreporting by actual height was slightly different, with a similar level of error for women taller than 160 cm, but greater overreporting of height for shorter women.

**Table 1:** Mean (SE) measured BMI, self-reported BMI, and difference across age and sex groups from cross-sectional surveys in 1995 and 2007-2008 (population-weighted data)

	1995				2007-2008			
Age group (years)	N	Measured BMI	Self-reported BMI	Difference in BMI*	N	Measured BMI	Self-reported BMI	Difference in BMI*
**Men**								
20-29	871	25.22 (0.17)	24.46 (0.16)	-0.76 (0.06)	704	25.66 (0.26)	25.30 (0.25)	-0.36 (0.07)
30-39	1065	26.63 (0.14)	25.60 (0.13)	-1.03 (0.05)	874	27.53 (0.22)	27.04 (0.22)	-0.49 (0.06)
40-49	904	27.27 (0.15)	26.09 (0.15)	-1.19 (0.06)	940	27.85 (0.22)	27.50 (0.21)	-0.36 (0.06)
50-59	731	27.70 (0.17)	26.33 (0.17)	-1.37 (0.07)	780	28.57 (0.21)	27.87 (0.21)	-0.70 (0.06)
60-69	649	27.77 (0.19)	26.25 (0.17)	-1.53 (0.07)	726	28.65 (0.22)	27.70 (0.21)	-0.96 (0.08)
70+	488	27.04 (0.18)	25.16 (0.18)	-1.88 (0.08)	543	27.94 (0.25)	26.70 (0.23)	-1.24 (0.10)
Overall	4708	26.77 (0.07)	25.59 (0.07)	-1.18 (0.03)	4567	27.60 (0.10)	26.99 (0.10)	-0.62 (0.03)
								
**Women**								
20-29	902	23.70 (0.21)	22.63 (0.17)	-1.07 (0.09)	650	24.68 (0.28)	24.17 (0.28)	-0.51 (0.07)
30-39	1070	25.13 (0.18)	23.97 (0.17)	-1.16 (0.06)	949	25.65 (0.19)	25.06 (0.19)	-0.59 (0.06)
40-49	982	26.16 (0.20)	24.93 (0.19)	-1.23 (0.06)	930	26.83 (0.22)	26.16 (0.23)	-0.67 (0.08)
50-59	760	27.23 (0.21)	25.81 (0.19)	-1.41 (0.06)	728	27.44 (0.28)	26.70 (0.27)	-0.75 (0.07)
60-69	667	27.32 (0.22)	25.56 (0.21)	-1.77 (0.08)	681	27.79 (0.24)	26.85 (0.22)	-0.94 (0.08)
70+	545	26.61 (0.22)	24.26 (0.20)	-2.35 (0.09)	636	26.93 (0.28)	25.65 (0.26)	-1.28 (0.11)
Overall	4926	25.76 (0.09)	24.36 (0.08)	-1.40 (0.03)	4574	26.44 (0.10)	25.70 (0.10)	-0.74 (0.03)

* Calculated from self-reported minus measured BMI; therefore, negative values indicate underreporting of BMI.

With regard to weight, the reporting error increases linearly (i.e., becomes more negative, indicating greater underreporting) with increasing actual weight of participants; this pattern is seen for both men and women and in both survey years (Figure 1).

**Figure 2:**
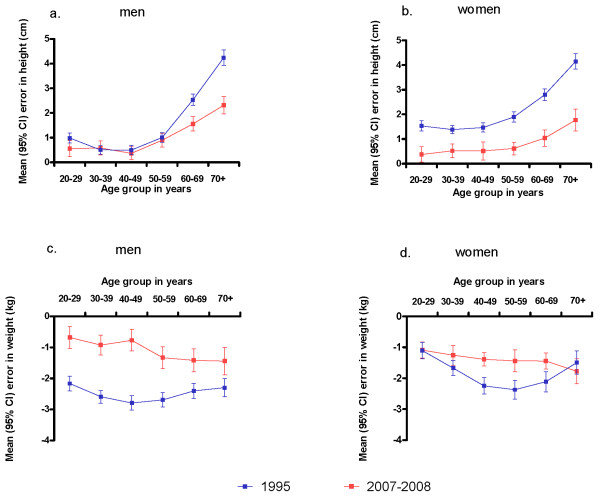
**Reporting error in height and weight (population-weighted) for men and women by age, determined from Australian NHS in 1995 and 2007-2008; a. height error in men; b. height error in women; c. weight error in men; d. weight error in women**.

Figure 2 shows the strong age dependency of reporting error in 1995 and in 2007-2008. In both surveys, men and women under 50 years had the smallest discrepancy between self-reported and actual height (approximately 0.5 cm in 2008), but the extent of misreport increased linearly with age above 50 years, rising to 2.3 cm for men and 1.8 cm for women over 70 years in 2007-2008.

Improved accuracy in reporting height between 1995 and 2008 was evident across most female age groups and also in the two oldest male age groups. Similarly, the underestimation in reporting weight was approximately linearly related with age in men and women, with less accuracy for older people. In the 13 years to 2008, men appeared to have had greater overall improvements than women in their reporting of weight.

### Determinants of reporting error

Age, sex, and actual BMI were highly significant determinants of reporting error in BMI (Table 2) with women underreporting their BMI by a larger amount than men. Underreporting of BMI also increased with age and with measured BMI. Of the socio-economic determinants investigated, only completion of year 12 at school was significant and indicated that people with more school education underreported BMI by a greater amount (approximately 0.2 units).

**Table 2:** Multiple regression analysis of error in reporting BMI in 2007-2008 using demographic and socio-economic predictor variables (population-weighted data)

	Coefficient (SE)	p-value
Measured BMI	-0.083 (0.005)	< 0.001
Age (years)	-0.013 (0.001)	< 0.001
Female	-0.216 (0.040)	< 0.001
SEIFA^a ^decile	-0.008 (0.011)	0.510
Born in Australia	0.036 (0.044)	0.415
Urban	0.040 (0.059)	0.497
Completed school year 12	-0.180 (0.045)	< 0.001
Completed tertiary qualification	0.020 (0.044)	0.653
Constant	2.329 (0.155)	< 0.001

^a ^**= **Socio-economic index for area

### Derivation and evaluation of correction equations

Linear regression models for predicting height, weight, and BMI error when only self-reported data are available are presented in Table 3. The pattern of misreporting height differed between men and women (Figure 1), and this was reflected in the different coefficients on self-reported height. Hence, we present separate equations for men and women's height error (Table 3). A spline function was used to capture the nonlinear effect of age, with different coefficients for under and over 60 years. The interpretation is that reporting error in height increases by 0.024 cm for every year of age up to 60 and by 0.1 cm for every year of age over 60. Regarding the prediction of weight error, men's and women's coefficients for age were not significantly different; hence, a single equation for weight error (both men and women) was derived with age and sex as covariates. Self-reported weight was not a significant covariate (p = 0.3) for prediction of weight error and was dropped from the regression. BMI error was predicted with separate equations for men and women, also using a spline function to model the nonlinear age effects observed.

**Table 3:** Linear regression models for reporting error in weight, height, and BMI, 2007-2008 Australian NHS (population-weighted data)

	Weight error	Height error		BMI error	
	Men and women N = 9141	Men N = 4567	Women N = 4574	Men N = 4567	Women N = 4574
	β (SE)	β (SE)	β (SE)	β (SE)	β (SE)
**Constant**	-0.3428 (0.150)^c^	-0.2201 (0.0196)^a^	-0.2918 (0.0225)^a^	-0.8088 (0.2537)^b^	-0.2856 (0.1584)^a^
**Age (years)**	-0.0152 (0.0029)^a^	-	-	-	-
**Age for each year up to age 60**	-	0.00023 (0.00005)^a^	0.00023 (0.00005)^a^	-0.0130 (0.0026)^a^	-0.0086 (0.0027)^b^
**Age for each year beyond age 60**	-	0.0010 (0.00013)^a^	0.0009 (0.00015)^a^	-0.0311 (0.0067)^a^	-0.0313 (0.0070)^a^
**Female**	-0.2973 (0.0952)^b^	-	-	-	-
**Self-reported height (meters)**	-	0.1225 (0.0109)^a^	0.1758 (0.0132)^a^	-	-
**Self-reported BMI**	-	-	-	0.0310 (0.0095)^b^	-
**R-squared**	0.0072	0.1064	0.1578	0.0392	0.0240

^a ^p < 0.001, ^b ^p < 0.01, ^c ^p < 0.05

### Internal validation

**Figure 3:**
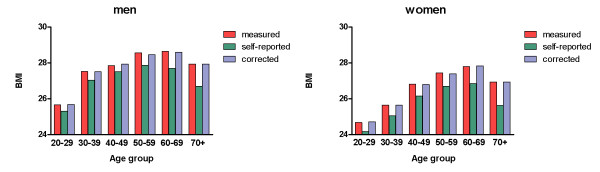
**Mean BMI by age and sex (population-weighted) using measured height and weight (NHS), self-reported height and weight (NHS) and corrected height and weight using correction equations applied to self-reported data**.

Corrected height can be calculated from self-reported height minus predicted height error. Using self-reported weight, height, and BMI and either of the two correction methods (correction of height and weight separately or direct correction of BMI), we determined corrected BMI for 9,141 survey participants and then examined the prevalence of underweight, normal weight, overweight, and obesity using measured, self-reported, and corrected data. Among women, mean BMI was predicted very well by both correction methods, and they were equally good at adjusting self-reported information to get estimates of the proportion of Australian women in each BMI category. However, for men, separate adjustment of height and weight gave closer estimates of the true percentage of overweight and obesity than through use of direct BMI correction. For example, the measured proportion of men that were classified as obese was 26.4%; use of correction equations applied to self-reported height and weight gave a value of 26.3%, while direct correction of self-reported BMI gave 28.9% (Table 4). A comparison of mean measured, self-reported, and corrected BMI by age and sex shown in Figure 3 demonstrates that the corrections were accurate across all age and sex strata.

**Table 4:** Measured, self-reported, and adjusted BMI (mean or percent [95% CI]), Australian NHS 2007-2008 (population-weighted data)

	Measured	Self-reported	Adjusted: method 1 (height and weight correction)	Adjusted: method 2 (BMI direct correction)
**Men (N = 4567)**				
**Mean BMI**	27.60 (27.41-27.80)	26.99 (26.79-27.18)	27.60 (27.40-27.79)	27.60 (27.41-27.79)
**BMI category (%)**				
**BMI < 18.5**	1.25 (0.68-1.81)	1.30 (0.74-1.85)	1.04 (0.55-1.53)	0.76 (0.30-1.23)
**BMI 18.5-24.99**	29.52 (27.73-31.30)	34.82 (32.80-36.85)	29.09 (27.25-30.94)	28.07 (26.25-29.89)
**BMI 25-29.99**	42.86 (40.78-44.95)	41.42 (39.48-43.36)	43.60 (41.70-45.49)	45.28 (43.34-47.23)
**BMI > 30**	26.37 (24.63-28.11)	22.46 (20.83-24.09)	26.27 (24.51-28.04)	28.88 (24.14-27.62)
				
**Women (N = 4574)**				
**Mean BMI**	26.44 (26.23-26.64)	25.70 (25.50-25.90)	26.42 (26.23-26.62)	26.43 (26.23-26.64)
**BMI category (%)**				
**BMI < 18.5**	2.47 (1.75-3.20)	3.30 (2.56-40.52)	1.92 (1.41-2.42)	1.76 (1.25-2.28)
**BMI 18.5-24.99**	43.90 (41.90-45.90)	49.75 (47.95-51.55)	44.22 (42.37-46.08)	44.35 (42.49-46.21)
**BMI 25-29.99**	31.25 (29.48-33.02)	27.97 (26.10-29.83)	32.29 (30.52-34.06)	32.19 (30.39-34.00)
**BMI > 30**	22.38 (20.69-24.08)	18.98 (17.44-20.52)	21.57 (19.96-23.17)	21.69 (20.14-23.25)

The overall level of misclassification of BMI categories was 19% through use of self-reported data; with the use of the correction equations, this was reduced to 16.6%. The greatest improvement in misclassification was in the obese category. Using self-reported data, 22.3% of obese people were not classified as such, but when the correction equations were applied this misclassification was reduced to 13.8% (Table 5).

**Table 5:** Individual patient misclassification (n [%]) of BMI categories using self-reported anthropometric data and corrected anthropometric data (correction method 1), 2007-2008 NHS

BMI category (measured data)	Misclassification (self-reported data)	Misclassification (corrected data)
**Underweight (BMI < 18.5)**	45/145 (31%)	65/145 (44.8%)
**Normal weight (BMI 18.5-24.99)**	294/3269 (9%)	487/3269 (14.9%)
**Overweight (BMI 25-29.99)**	891/3439 (25.9%)	649/3439 (18.9%)
**Obese (BMI > 30)**	510/2288 (22.3%)	315/2288 (13.8%)
**All**	1740/9141 (19.0%)	1516/9141 (16.6%)

## Discussion

In this study, we examined the bias in reporting of height and weight in Australia using large nationally representative population health survey data. Consistent with a systematic review in 2007 [2] and an earlier study in Australia [7], we found a tendency for men and women to overestimate their height and to underestimate their weight, thus resulting in an underestimation of BMI. The direction of the bias was consistent with that reported in previous studies [2,7,8].

We found that the bias in self-reported BMI (derived from height and weight) has decreased in Australia between 1995 and 2008. Examination of age- and gender-specific effects suggested that the decline is due to more accurate reporting of both height and weight across most sectors of the population. The exception was in the reporting of height among young men, whose average reporting error was similar in 1995 and 2007-2008 at approximately 0.5 cm, an amount consistent with the rounding up of height in centimeters.

Since reporting error is highly dependent on measured BMI, and measured BMI has increased in Australia between 1995 and 2008, we might have expected reporting error to have increased over this period. Instead the converse has been found - the average reporting error in BMI in 1995 was -1.2 units for men and -1.4 for women; in 2007-2008 it had decreased to -0.6 and -0.7 units, respectively.

Using 2007-2008 data, we found the major determinants of the reporting bias to be age, sex, measured BMI, and school education level. Neither the increasing trend in BMI in Australia nor the increased proportion of participants to have completed year 12 at school can explain the diminishing reporting bias between 1995 and 2008, since the signs of both these coefficients were negative.

The diminishing reporting bias observed in this study strongly suggests that there has been an improvement in awareness of personal height and weight across the Australian adult population. Obesity is a National Health Priority Area [17] in Australia, and in recent years there has been increased coverage of obesity issues in the media [18], including high profile public health campaigns, such as the "Measure Up" campaign [19].

There are very few studies across different countries with which to compare our results. However, the observed diminishing bias in Australia is contrary to a recent study that found reporting error in the United States remained constant between 1976 and 2005 but increased in Canada between 1986 and 2005 [4]. There may be differences in the temporal change in reporting bias across countries, in the same way that reporting bias itself may be affected by ethnicity or country-specific factors [20]. Indeed, country-specific factors may contribute to the large variation in reporting bias among studies included in a recent systematic review [2].

Another factor that may affect the change in reporting bias in different countries is the preferred units in which participants give their self-reported height and weight. Australia moved to a metric system in 1976, and participants in the earlier survey (and older participants in particular) are more likely to have reported height and weight in imperial units, which may be subject to different rounding than if metric units were used [21]. By comparison, in the United States, which has retained the imperial system of measurement, there was no change in reporting bias observed in national surveys between 1976 and 2005.

Despite the improvement in accuracy of reporting BMI, estimates of the prevalence of obesity in Australia in 2007-2008 from self-reported data were underestimated by approximately 4%. This underestimation is driven mainly by older age groups (> 60 years) whose reporting of height, in particular, was much less accurate than younger people.

Using 2007-2008 data we derived two correction methods to adjust for the reporting bias, using age, sex, and self-reported data as covariates. In internal validation, we showed that use of separate correction equations for height and weight were able to provide accurate estimates of the population prevalence of each BMI category and appeared to be more accurate than directly correcting self-reported BMI. The equations for height explained a much greater proportion of the measurement error than those for weight. We have been unable to test the correction equations in an independent population, but if collection of self-reported height and weight is similar to NHS methodology and if the age of the participants is known, we would expect the correction equations to perform very well.

The major strength of this study is that it is based on very recent large nationally representative surveys, in which the use of person weights allows us to infer the results to the entire adult population of Australia. Additionally, we have examined reporting bias across different age and sex strata and have been able to derive correction equations incorporating age and gender. Although there has been some debate as to whether correction equations are useful and reliable [22], our results suggest that when height and weight are adjusted separately, the corrected estimates of obesity and overweight prevalence at a population level and for age/sex subgroups are very close to those determined from measured data. A spreadsheet version of the correction equations, implemented in Excel, may be downloaded from http://www.health.usyd.edu.au/heconomics/resources/supplementary.php.

There are some limitations to our study. Our results may be affected by slight differences in collection methods between surveys in 1995 and 2007-2008. For example, in the 1995 NNS survey, measured data were determined up to three weeks after the determination of self-reported data from the NHS [23], whereas in 2007-2008, measurements were taken on the same day as self-reported information was provided.

In both surveys, participants were supplied with information prior to interview stating that there would be a request to take height, weight, hip, and waist measurements. Hence, the lower reporting bias observed in 2007-2008 compared to 1995 cannot be attributed to differences in knowledge that physical measurements would be taken.

With respect to determinants of reporting bias, we were unable to investigate the effect of race or ethnicity (which was significant in US populations [8]) as ethnicity is not collected in the NHS. Finally, the correction equations for height and weight were accurate in their predictions of true overweight and obesity prevalence at a population level, but at an individual patient level, there is limited improvement in misclassification of BMI category, and hence they should be used with caution for individual prediction.

There are implications of the observed change in reporting bias in Australia for transferability of the results and for projections of trends in obesity. First, our correction equations, although valid for current (2007-2008) data, may not be valid for older surveys or for surveys in the future. This could be investigated when the next NHS results become available in 2011. Second, predictions of future overweight and obesity in Australia from extrapolation of parametric equations derived from past trends in self-reported data [24] will be inappropriate because change in reporting bias will affect the apparent increase in self-reported obesity. For example, using measured data, the point increase in population obesity in Australia between 1995 and 2008 was 6.1% (from 18.7% to 24.8%), while the self-reported data suggested it was 10.3% (from 11.1% to 21.4%). Hence, part of the apparent increase in obesity prevalence based on self-reported data is due to the decline in reporting bias. Rising obesity rates in Australia [25] are a major public health concern, but tracking trends in obesity and overweight using self-reported data may be quite misleading, if, as we have shown here, the reporting bias has changed substantially over time.

## Conclusions

We have developed correction equations to adjust for the bias in self-reported height and weight, which provide accurate estimates of mean BMI and obesity prevalence by age and gender subgroups. Self-reporting bias in anthropometric data has diminished in Australia between 1995 and 2008; hence, researchers should be careful to use the most appropriate correction algorithms when estimating population obesity prevalence and trends from self-reported data. The discrepant findings in the temporal change in reporting bias observed in the US, Canada, and Australia warrant further investigation. Comparison of results of similar health surveys from other countries would be invaluable.

## List of abbreviations

BMI: body mass index; ABS: Australian Bureau of Statistics; NNS: National Nutritional Survey; NHS: National Health Survey; CURF: confidential unit record file; SEIFA: socio-economic index for area.

## Competing interests

The authors declare that they have no competing interests.

## Authors' contributions

AH conceived the study, participated in its design and coordination, performed the statistical analysis, and drafted the manuscript. PC participated in the design of the study and writing of the manuscript. TL contributed to the statistical analysis and literature search and coordination. All authors read and approved the final manuscript.
